# Utilization and Safety of Percutaneous Lung Biopsy: A 10-Year Nationwide Population-Based Study

**DOI:** 10.3390/ijerph16081316

**Published:** 2019-04-12

**Authors:** Chun-Ku Chen, Hsiao-Ting Chang, Yu-Chun Chen, Shu-Chiung Chiang, Hsiao-Ping Chou, Tzeng-Ji Chen

**Affiliations:** 1 Department of Radiology, Taipei Veterans General Hospital, Taipei 11217, Taiwan; ckchen@vghtpe.gov.tw (C.-K.C.); gutmomo@gmail.com (H.-P.C.); 2 Department of Radiology, Faculty of Medicine, School of Medicine, National Yang-Ming University, Taipei 11221, Taiwan; 3 Institute of Clinical Medicine, School of Medicine, National Yang-Ming University, Taipei 11221, Taiwan; 4 Institute of Hospital and Health Care Administration, School of Medicine, National Yang-Ming University, Taipei 11221, Taiwan; yuchn.chen@gmail.com (Y.-C.C.); scchiang0g@gmail.com (S.-C.C.); 5 Department of Family Medicine, Taipei Veterans General Hospital, Taipei 11217, Taiwan; htchang2@vghtpe.gov.tw; 6 Department of Family Medicine, Faculty of Medicine, School of Medicine, National Yang-Ming University, Taipei 11221, Taiwan; 7 Division of Radiology, Yonghe Cardinal Tien Hospital, New Taipei City 23445, Taiwan

**Keywords:** lung biopsy, procedure safety, utilization

## Abstract

Percutaneous lung biopsy is a technique used for sampling peripherally located lung masses and has been gaining in popularity. However, its exact utilization is unknown, and its safety has not been well studied. The current study aimed to assess the trend of utilization and study the safety of this procedure. Using the National Health Insurance Research Database, we retrospectively determined the total number of procedures that were performed on subjects older than 20 years between 2001 and 2010. We also estimated the rates of major complications, such as pneumothorax, requiring intercostal drainage. A total of 630 percutaneous biopsies were performed in 2001, while 3814 were performed in 2010, representing a 6.1-fold increase. The compound annual growth rate was 22.1%. The number of hospitals that performed the procedure increased from 55 to 99. Pneumothorax requiring drainage occurred in 1.5% of the procedures. The factors associated with a higher complication rate included male gender, chronic obstructive pulmonary disease, rural hospital, and low-volume hospital. Percutaneous lung biopsies are a relatively safe procedure, and their performance has been rapidly increasing. The number of procedures performed by a hospital was associated with the complication rate.

## 1. Introduction

Since its introduction, percutaneous lung biopsy has become an important method of obtaining lung tissue to characterize focal lung diseases such as lung cancer [1]. This technique does not require bronchoscopy via the pharynx and may be better tolerated by some patients. Using a cutting needle, a percutaneous lung biopsy is able to yield a larger specimen than that by bronchoscopic biopsy, thus, providing better diagnostic accuracy [2]. 

When compared with percutaneous lung biopsy, the bronchosopic procedure needs a preprocedural local spray of anesthetic agents to the throat, and some waiting time for the sprayed agents to be effective; moreover, for lesions that are not adjacent to the large airway or for peripheral lung lesions, additional fluoroscopic guidance may be needed, which would take more time with the addition of the operation of fluoroscopy. Modern techniques, such as electromagnetic navigation, can navigate the bronchoscopy and the devices to biopsy the peripheral pulmonary nodules via virtual three-dimensional images; however, the need to attach the sensor and load images in the planning phase would take even more time [3]. Regarding cost, the electromagnetic navigational bronchoscopic biopsy for the lung nodule has a higher cost than computed tomography (CT)-guided lung biopsy [4]. Bronchoscopy is also known to cause hypoxemia [5]. While percutaneous lung biopsy has several advantages over bronchoscopic lung biopsy as mentioned earlier, nevertheless, due to the percutaneous nature of this approach, the pleura will be punctured and pneumothorax is more likely to occur with this approach when compared with bronchoscopic biopsies [4].

Due to the rapidly increasing need for the histological typing of lung tissue [6,7], the need for percutaneous lung biopsies may continue to increase due to its suitability for the subtyping and genotyping of lung cancer [8], and the increasing use of CT scans for percutaneous lung biopsy could lower the availability of CT scanners for other purposes [9]. As percutaneous lung biopsies are most commonly performed with CT guidance, understanding their utilization trends may provide a reference regarding resource and equipment allocation for hospital administrators. CT advancements have enabled the detection of smaller nodules [10,11], which make percutaneous biopsies technically more difficult [12], nevertheless, lung cancers of a small size had good prognosis [13]. Personnel training would be important if the utilization of percutaneous lung biopsy rapidly increases.

In the current study, we aimed to evaluate the utilization trends of percutaneous lung biopsy and estimate its safety profile with the aim of identifying the associated risk factors for complications.

## 2. Materials and Methods 

### 2.1. Database

This study used the National Health Insurance Research Database, which consists of de-identified secondary data derived from the claims and registry data of the Taiwan National Health Insurance (NHI) Program beginning in 1995. Under this program, the Bureau of National Health Insurance (BNHI) has enrolled 99.5% of the nation’s inhabitants [14,15]. Annually, the Taiwan National Health Research Institute (NHRI) de-identifies and publishes the registry and claims data released by the BNHI. Before publication, the NHRI replaces all information about the beneficiaries, surgeons, and institutions with random combinations of characters to protect their privacy. Through this method, the NHRI not only makes it feasible to identify claims related to the same beneficiary, surgeon, or institution but it also makes the identification of a specific beneficiary, surgeon, or institution impossible. This study was approved by the Institutional Review Board of Taipei Veterans General Hospital (VGHIRB No.2013-02-015BC). Informed consent was waived as the database is de-identified and specific individuals could not be identified.

### 2.2. Identification of Percutaneous and Bronchoscopic Lung Biopsy Cases

We identified all records with a code of percutaneous lung biopsy (ICD-9-CM procedure code 33.26) or bronchoscopic lung biopsy (ICD-9-CM procedure code 33.27) from the claims database between 1 January 2001 and 31 December 2009.

### 2.3. Demographics and Comorbid Conditions

The ages of the subjects at the time of biopsy were identified, and the insurance levels were used as a proxy for socioeconomic status. All of the subjects’ income levels were grouped into three categories of premiums paid (NTD$ ≥40,000, 20,000–39,999, 1–19,999). In the Taiwan NHI, health insurance premiums are primarily determined based on the insured wage and premium rate, thus, a higher premium implies a higher income. 

The urbanization of the location of the NHI registration is recorded as a proxy of residential urbanization. Taiwan is divided into seven urbanization levels, with level 1 defined as the “most urbanized” and level 7 defined as the “least urbanized”. We designated level 1 as the metropolitan area; levels 2–3 as the urban area, and levels 4–7 as the rural area.

We also identified the baseline comorbidities associated with a complicated lung biopsy as follows: chronic obstructive pulmonary disease (COPD) (ICD-9-CM code: 490–496.x), renal failure (530.0–530.9), chronic liver disease and cirrhosis (571.x), coagulation defects and hemorrhagic conditions (286–287.x), ischemic heart disease (410–414.x), and stroke (430.1x, 433.x1, 434.x, 436.x).

### 2.4. Hospital Characteristics

The hospitals performing the biopsies were identified as medical centers, regional hospitals, or local hospitals. The medical centers were tertiary referral centers, and because the volume of procedures performed outside of the medical centers was small, we combined regional and local hospitals into a non-medical center category. The number of percutaneous lung biopsies performed was divided by the median value and rounded to the nearest 100 biopsies per year. The urbanization of the hospital was designated by the same method as that used to designate residence urbanization, as described in the previous section.

### 2.5. Outcomes

Perioperative complications were defined as complications if they occurred within 30 days of the biopsy procedure. Pneumothorax requiring drainage was defined as a diagnostic code of pneumothorax (ICD-9-CM code: 512.x) and the insertion of a chest tube (ICD-9-CM procedure code: 34.04) in the same visit. Hemoptysis (ICD9-CM code 786.3), cardio-pulmonary resuscitation (ICD-9-CM procedure code: 99.60), surgery for hemorrhage control (ICD-9-CM procedure code: 39.98), and exploratory thoracotomy (ICD-9-CM procedure code: 34.02) were all searched.

### 2.6. Statistical Analysis

Data management and computing were performed with Microsoft SQL Server 2012 (Microsoft Corporation, Redmond, WA, USA). Statistical analysis was performed with SPSS (Version 20.0, IBM, Armonk, NY, USA). The compound annual growth rate (CAGR) was calculated to represent the trend of utilization by the following equation:

*CAGR* = ((*N*_2010_/*N*_2001_)^1/9)) − 1

Complication rates were computed as the number of each event divided by the number of procedures performed during the study period. If a subject had more than one lung biopsy procedure, only the first procedure was included. We excluded any procedures associated with tuberculosis or pleurisy, as the insertion of an intercostal catheter is likely and could lead to an overestimation of the complications. Categorical variables were compared using the χ^2^ test or Fisher’s exact test, as appropriate, and continuous variables were compared using the Student’s *t*-test. To compare the complications, if there were multiple biopsies for a single subject, only the first biopsy was included. Associations between the hospital characteristics and the outcomes were assessed by multivariate logistic regression. The trends of percutaneous and bronchoscopic lung biopsies were analyzed by the χ^2^ test for linear trends. P < 0.05 (two-sided) was considered significant.

## 3. Results

### 3.1. Overall Utilization

**Figure 1:**
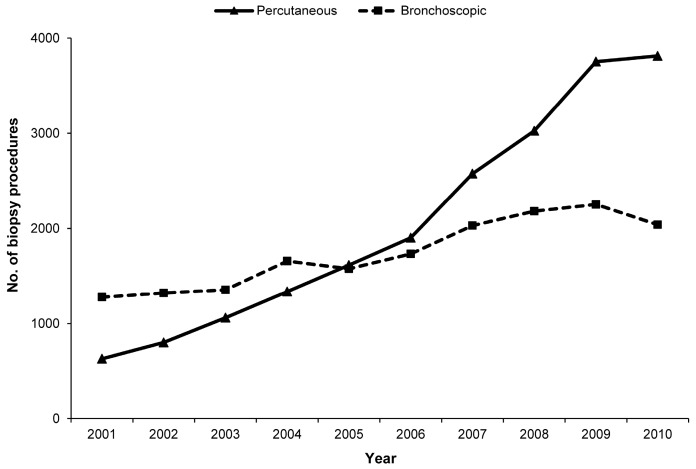
Trends in the utilization of lung biopsies. The use of percutaneous lung biopsies increased rapidly between 2001 and 2010.

A total of 20,518 percutaneous lung biopsy procedures were performed in 19,330 subjects. The total number of percutaneous lung biopsy procedures increased from 630 in 2001 to 3814 in 2010, and the number of hospitals performing percutaneous biopsies increased from 55 in 2001 to 99 in 2010. There was a 6.1-fold increase in percutaneous lung biopsies between 2001 and 2010. The CAGR for percutaneous lung biopsies was 22.1% during the same period. The number of bronchoscopic procedures was 1278 in 2001 and peaked in 2009 with 2252 procedures (but fell to 2040 in 2010). The number of hospitals performing bronchoscopic lung biopsies was 67 in 2001, with a slight increase to 90 in 2010 (Figure 1). The ratio of percutaneous to bronchoscopic lung biopsies showed an increasing trend from 2001 to 2010.

**Figure 2:**
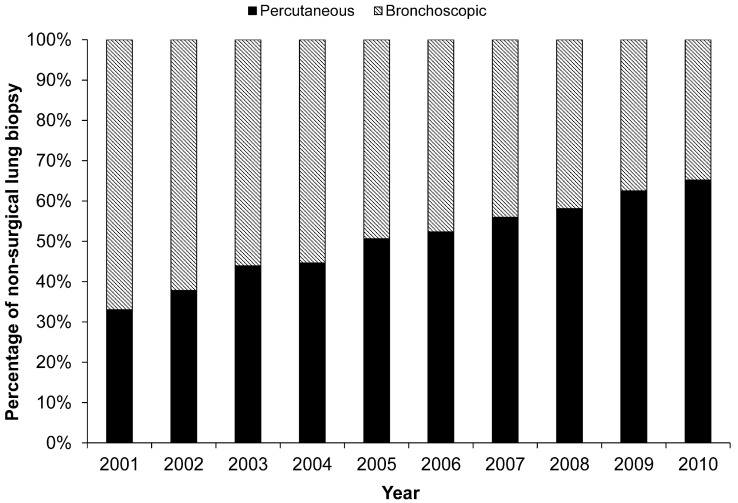
Trends in the ratio of percutaneous lung biopsies to non-surgical lung biopsies. In 2001, only 33% of the lung biopsies were percutaneous, whereas in 2010, percutaneous biopsies constituted 65% of all lung biopsies.

Percutaneous lung biopsies constituted 33% and 65% of all non-surgical lung biopsies in 2001 and 2010, respectively (Figure 2). Significant differences were found in the trends of utilization between percutaneous lung biopsy and bronchoscopic biopsy (*p* < 0.001).

### 3.2. Utilization Based on Hospital Characteristics

Approximately 38% of the percutaneous lung biopsies were performed in the Taipei metropolitan region, whereas only 24% of the bronchoscopic lung biopsies were performed in the same area. Northern Taiwan hospitals performed only 7% of the percutaneous lung biopsies, but performed 23% of the bronchoscopic lung biopsies. The ratio of percutaneous to bronchoscopic biopsies was close to 1:1 in the Central, South, and Kaohsiung Metropolitan areas (Table 1).

Medical centers (tertiary referral centers) performed 445 biopsies in 2001 and 2398 in 2010, whereas non-medical centers performed 185 in 2001 and 1416 in 2010. The CAGRs for medical centers and regional/local hospitals were 20.6 and 25.4%, respectively. There was a difference in the trends of percutaneous lung biopsy utilization between the different hospital levels (*p* < 0.001).

The use of percutaneous lung biopsy procedures differed between hospital urbanization categories, with CAGRs of 16.0%, 27.4%, and 21.9% for metropolitan, urban, and rural hospitals, respectively.

### 3.3. Complications

Among the 19,330 subjects who underwent percutaneous lung biopsies, 1112 subjects were associated with tuberculosis or pleurisy and were excluded from the analysis of complications. From the total of 18,218 percutaneous lung biopsies that were included in the analysis, the rate of intercostal drainage catheter use due to pneumothorax was 1.5%. There was a 0.1% overall exploratory thoracotomy rate and a 0.2% overall cardiopulmonary resuscitation rate (Table 2). Nevertheless, major complication-free outcomes (no cardiopulmonary resuscitation, no exploratory thoracotomy, no chest tube insertion for pneumothorax, no operation for hemorrhage) were noted in 98.1% of percutaneous lung biopsy procedures.

We further analyzed the factors associated with major complications (Table 3). Male subjects, subjects older than 65 years, subjects who underwent percutaneous lung biopsies in a hospital in a rural area, subjects with COPD, renal failure, and subjects who had undergone percutaneous biopsy at a low-volume hospital or non-medical center were more likely to have suffered a pneumothorax requiring intercostal drainage. Male subjects, elderly subjects, and subjects who received a biopsy in a rural, low-volume, or non-medical center hospital were more likely to suffer hemoptysis. Subjects who were male and elderly, had COPD, had lower income, had a stroke, or underwent procedures in a low-volume hospital or non-medical center hospital had higher cardiopulmonary resuscitation rates. Subjects who had coagulation disorders and who underwent percutaneous lung biopsies in a rural hospital or a non-medical center were more likely to have an exploratory thoracotomy.

Bronchoscopic biopsies were associated with a 98.1% major complication-free rate, 0.3% cardiopulmonary resuscitation rate, 0.1% exploratory thoracotomy rate, and 1.1% rate of intercostal drainage for pneumothorax. We found no significant difference in the major complication-free rates between bronchoscopy and percutaneous lung biopsy (*p* = 0.829).

The results of the multivariate analysis are shown in Table 4. Male gender, COPD, rural hospital, and low-volume hospital were associated with pneumothorax requiring drainage. Male gender, elderly, rural hospital, low-volume hospital, and non-medical center were associated with hemoptysis. Non-medical centers were associated with cardiopulmonary resuscitation. Coagulation disorders were associated with exploratory thoracotomy.

## 4. Discussion

The present study demonstrated a rapid increase in the utilization of percutaneous lung biopsies during 2001–2010. The complication rate observed was reasonable, and there were no differences in the major complication rates between percutaneous lung biopsies and bronchoscopic lung biopsies.

The use of bronchoscopic lung biopsies increased slowly from 2001 to 2009, but decreased in 2010, whereas the use of percutaneous lung biopsies increased rapidly throughout the same period. The observed increasing popularity of percutaneous lung biopsies is consistent with the findings of a previous study [16]. 

The previously reported rate of pneumothorax requiring drainage in percutaneous lung biopsies was 0 to 6.6% [17,18,19]. The current study showed that the rate of pneumothorax requiring drainage was 1.5%, which is slightly lower than some previously reported values [18]. The fewer passages through the pleural surface may have decreased the probability of a significant pneumothorax. Moore et al. found that if only one pleural puncture were used, the chest tube placement rate was 0.4% when compared with an overall chest tube placement rate of 1.6%. [20] However, Ohno et al. found that a single puncture resulted in no chest tube insertion, whereas three punctures in a lung resulted in a chest tube insertion rate of 50%. Similarly, a study using a coaxial needle revealed no chest tube insertion rate in cases of pneumothorax [17]. Assuming that coaxial usage was routine in our study due to the fact that coaxial needles are reimbursed by the BNHI and no subjects had to pay out-of-pocket costs, the rate of pneumothorax requiring drainage was reasonably accurate.

The possible reason for the increased popularity of percutaneous lung biopsies was possibly due to the accuracy of percutaneous biopsies. A previous report found that percutaneous CT-guide biopsy had a diagnostic yield of 93%, higher than that of bronchoscopy biopsy at 75%. Additionally, the proportion of the more commonly regarded centrally located squamous cell carcinoma has decreased over the past 30 years, from 49.6% to 34.8% [21]. Moreover, traditionally recognized centrally located squamous cell carcinoma has been reported to consist of more than 62% of squamous cell carcinoma [22].

The strengths of our study include its use of a large, population-based sample that is representative of the national population of Taiwan, which was made possible by accessing data from a compulsory, government-run, centralized, single-payer universal health insurance system covering treatment for all major medical or surgical events. Healthcare services provided by overseas providers are also covered by the system. Loss of follow-up is, therefore, only possible if users do not seek medical attention, suggesting that our estimates of incidence are likely to be accurate and representative of the national population.

This study has several limitations. First, it is unclear whether pneumothorax requiring drainage is a complication of or related to percutaneous lung biopsy, as there were no medical charts available for review. Nevertheless, it is less likely that a biopsy would have been performed to investigate a pneumothorax when compared with the probability that a pneumothorax would be an unintentional complication of a biopsy. Moreover, we excluded the common reason, which is pleural effusion for intercostal drainage insertion. Thus, we considered this issue to be at least partially addressed. Second, the rate of pneumothorax may have been underestimated. Our standards required a concurrent diagnosis of pneumothorax in addition to the performance of an intercostal drainage procedure to qualify as a pneumothorax complication. However, these stipulations mean that minor cases of pneumothorax may have been missed.

## 5. Conclusions

In conclusion, percutaneous lung biopsy utilization increased rapidly between 2001 and 2010. This procedure is relatively safe, although the volume of procedures performed by a given hospital was associated with the complication rate.

## Figures and Tables

**Table 1:** Utilization and hospital characteristics.

Hospital Characteristics	Year										Total
	2001	2002	2003	2004	2005	2006	2007	2008	2009	2010	
Total No.	630	802	1061	1337	1616	1902	2576	3026	3754	3814	20,518
Hospital characteristics											
Urbanization											
Metropolitan	314	385	481	607	663	724	928	898	1163	1192	7355
Urban	257	343	484	573	676	881	1349	1846	2280	2271	10,960
Rural	59	74	96	157	277	297	299	282	311	351	2203
Geographical Region											
Taipei Metropolitan	270	300	365	517	540	693	923	1153	1473	1516	7750
Northern	93	109	130	171	214	144	189	128	189	163	1530
Central	32	66	115	142	186	247	402	551	679	698	3118
Kaohsiung Metropolitan	112	179	265	322	415	462	528	580	691	716	4270
Southern	86	96	128	146	193	319	449	515	618	606	3156
Eastern	37	52	58	39	68	37	85	99	104	115	694
Referral status											
Medical Center	445	587	774	918	1019	1157	1684	1957	2414	2398	13,353
Non-medical center	185	215	287	419	597	745	892	1069	1340	1416	7165

**Table 2:** Complications of percutaneous lung biopsy.

Complications (*N* = 18,218)	No.	(%)
Pneumothorax with drainage	274	1.5
Hemoptysis	290	1.6
Cardiopulmonary resuscitation	41	0.2
Exploratory thoracotomy	17	0.1

**Table 3:** Subjects and hospital characteristics and complications.

Characteristics	Total No.	Pneumothorax with Drainage			Hemoptysis			Cardiopulmonary Resuscitation			Exploratory Thoracotomy		
		*n*	(%)	*p*-Value	*n*	(%)	*p*-Value	*n*	(%)	*p*-Value	*n*	(%)	*p*-Value
Demographics													
Gender				<0.001			0.008			0.03			0.27
Female	6626	65	(1.0)		84	(1.3)		8	(0.1)		4	(0.1)	
Male	11,592	209	(1.8)		206	(1.8)		33	(0.3)		13	(0.1)	
Age				0.006			0.008			0.003			0.70
<65	7728	94	(1.2)		145	(1.9)		8	(0.1)		8	(0.1)	
≥65	10,490	180	(1.7)		145	(1.4)		33	(0.3)		9	(0.1)	
Insurance amount				0.15			0.29			0.02			0.68
<20,000	3890	48	(1.2)		57	(1.5)		15	(0.4)		4	(0.1)	
20,000–39,999	10,363	171	(1.7)		178	(1.7)		23	(0.2)		8	(0.1)	
≥40,000	3965	55	(1.4)		55	(1.4)		3	(0.1)		5	(0.1)	
Subject Urbanization				0.49			0.14			0.04			0.19
Metropolitan	5057	71	(1.4)		79	(1.6)		11	(0.2)		5	(0.1)	
Urban	7796	127	(1.6)		111	(1.4)		11	(0.1)		4	(0.1)	
Suburb/Rural	5365	76	(1.4)		100	(1.9)		19	(0.4)		8	(0.1)	
Comorbidities													
Cirrhosis				0.91			0.30			0.05			0.21
No	16,656	250	(1.5)		270	(1.6)		34	(0.2)		17	(0.1)	
Yes	1562	24	(1.5)		20	(1.3)		7	(0.4)		0	(0.0)	
Coagulation disorders				0.61			0.81			0.13			0.03
No	17,942	269	(1.5)		287	(1.6)		39	(0.2)		15	(0.1)	
Yes	276	5	(1.8)		3	(1.1)		2	(0.7)		2	(0.7)	
COPD				<0.001			0.873			0.02			0.12
No	14,704	174	(1.2)		233	(1.6)		27	(0.2)		11	(0.1)	
Yes	3514	100	(2.8)		57	(1.6)		14	(0.4)		6	(0.2)	
Diabetes Mellitus				0.05			0.05			0.34			>0.99
No	14,437	230	(1.6)		243	(1.7)		30	(0.2)		14	(0.1)	
Yes	3781	44	(1.2)		47	(1.2)		11	(0.3)		3	(0.1)	
Hypertension				0.87			0.16			0.98			0.11
No	11,587	173	(1.5)		196	(1.7)		26	(0.2)		14	(0.1)	
Yes	6631	101	(1.5)		94	(1.4)		15	(0.2)		3	(0.0)	
Ischemic heart disease				0.17			0.14			0.47			>0.99
No	15,803	230	(1.5)		260	(1.6)		34	(0.2)		15	(0.1)	
Yes	2415	44	(1.8)		30	(1.2)		7	(0.3)		2	(0.1)	
Renal failure				0.02			0.11			0.50			0.62
No	17,202	250	(1.5)		280	(1.6)		38	(0.2)		17	(0.1)	
Yes	1016	24	(2.4)		10	(1.0)		3	(0.3)		0	(0.0)	
Stroke				0.72			0.04			0.02			0.63
No	16,986	254	(1.5)		279	(1.6)		34	(0.2)		17	(0.1)	
Yes	1232	20	(1.6)		11	(0.9)		7	(0.6)		0	(0.0)	
Hospital characteristics													
Hospital Urbanization				0.001			<0.001			0.06			0.04
Metropolitan	6567	77	(1.2)		98	(1.5)		13	(0.2)		4	(0.1)	
Urban	9739	152	(1.6)		122	(1.3)		19	(0.2)		8	(0.1)	
Suburb/Rural	1912	45	(2.4)		70	(3.7)		9	(0.5)		5	(0.3)	
Volume (100)				0.007			<0.001			0.04			0.02
Low	10,862	185	(1.7)		230	(2.1)		31	(0.3)		15	(0.1)	
High	7356	89	(1.2)		60	(0.8)		10	(0.1)		2	(0.03)	
Hospital grade				0.04			<0.001			<0.001			0.01
Medical center	12,013	165	(1.4)		130	(1.1)		15	(0.1)		6	(0.05)	
Non-medical center	6205	109	(1.8)		160	(2.6)		26	(0.4)		11	(0.2)	

**Table 4:** Multivariate analysis for factors associated with complications.

Characteristics	Pneumothorax with Drainage			Hemoptysis			Cardiopulmonary Resuscitation			Exploratory Thoracotomy		
	Odds Ratio	95% CI	*p*-Value	Odds Ratio	95% CI	*p*-Value	Odds Ratio	95% CI	*p*-Value	Odds Ratio	95% CI	*p*-Value
Demographics												
Male gender	1.56	(1.17–2.08)	0.002	1.48	(1.14–1.92)	0.003	1.99	(0.90–4.38)	0.09	1.66	(0.53–5.24)	0.39
Age ≥ 65	1.18	(0.90–1.56)	0.23	0.68	(0.53–0.88)	0.003	2.17	(0.96–4.92)	0.06	0.70	(0.24–2.00)	0.50
Insurance amount			0.27			0.30			0.09			0.52
<20,000	1.00	(reference)		10.00	(reference)		10.00	(reference)		1.00	(reference)	
20,000–39,999	1.31	(0.94–1.81)	0.11	1.18	(0.87–1.60)	0.28	0.62	(0.32–1.20)	0.15	0.78	(0.23–2.63)	0.69
≥40,000	1.25	(0.83–1.86)	0.29	0.95	(0.65–1.40)	0.80	0.27	(0.08–0.97)	0.04	1.56	(0.39–6.28)	0.54
Subject Urbanization			0.07			0.50			0.30			0.33
Rural	1.00	(reference)		10.00	(reference)		10.00	(reference)		1.00	(reference)	
Urban	1.38	(0.97–1.97)	0.08	1.12	(0.80–1.56)	0.50	0.98	(0.43–2.23)	0.95	0.93	(0.26–3.31)	0.91
Metropolitan	1.42	(1.05–1.92)	0.02	0.94	(0.70–1.25)	0.65	0.56	(0.26–1.22)	0.14	0.41	(0.11–1.44)	0.16
Comorbidities												
Cirrhosis	0.90	(0.59–1.39)	0.65	0.76	(0.47–1.20)	0.24	1.66	(0.71–3.85)	0.24	0.00	(0.00–Inf)	0.99
Coagulation disorders	1.12	(0.46–2.77)	0.80	0.72	(0.23–2.27)	0.57	3.03	(0.71–12.96)	0.14	13.99	(3.05–64.08)	0.001
COPD	2.14	(1.64–2.81)	<0.001	1.03	(0.75–1.40)	0.86	10.36	(00.69–20.71)	0.38	2.42	(0.82–7.11)	0.11
Diabetes Mellitus	0.70	(0.50–0.98)	0.04	0.77	(0.56–1.07)	0.11	10.27	(00.62–20.61)	0.52	1.11	(0.31–4.06)	0.87
Hypertension	0.93	(0.70–1.22)	0.58	1.07	(0.81–1.40)	0.64	00.70	(00.34–10.43)	0.33	0.39	(0.10–1.50)	0.17
Ischemic heart disease	1.01	(0.71–1.43)	0.96	0.88	(0.59–1.32)	0.54	00.94	(00.39–20.24)	0.88	1.26	(0.26–6.18)	0.78
Renal failure	1.40	(0.91–2.16)	0.13	0.58	(0.31–1.11)	0.10	00.86	(00.26–20.88)	0.81	0.00	(0.00–Inf)	0.99
Stroke	0.96	(0.60–1.54)	0.86	0.56	(0.30–1.04)	0.07	20.12	(00.89–50.06)	0.09	0.00	(0.00–Inf)	0.99
Hospital characteristics												
Hospital Urbanization			0.002			0.001			0.67			0.71
Rural	1.00	(reference)		1.00	(reference)		10.00	(reference)		10.00	(reference)	
Urban	0.43	(0.27–0.69)	<0.001	0.66	(0.44–0.98)	0.04	10.35	(00.48–30.81)	0.57	0.50	(0.09–2.71)	0.42
Metropolitan	0.57	(0.38–0.86)	0.007	0.52	(0.37–0.74)	<0.001	00.96	(00.40–20.32)	0.93	0.65	(0.18–2.37)	0.51
Volume			0.04			0.001			0.47			0.16
Low	1.00	(reference)		1.00	(reference)		10.00	(reference)		10.00	(reference)	
High	0.72	(0.52–0.98)		0.55	(0.39–0.78)		10.49	(00.50–40.42)		0.29	(0.05–1.60)	
Hospital grade			0.15			0.007			0.01			0.50
Non-medical center	1.00	(reference)		1.00	(reference)		1.00	(reference)		10.00	(reference)	
Medical center	1.29	(0.92–1.83)		0.64	(0.46–0.88)		0.26	(0.09–0.74)		0.63	(0.17–2.40)	

## References

[1] Haaga J.R., Alfidi R.J. (1976). Precise biopsy localization by computer tomography. Radiology.

[2] Kardos L., Nagy E., Morvay Z., Fuzesi E., Furak J., Tiszlavicz L., Horvath I., Palko A. (1999). Value of CT-guided biopsy compared to fluoroscopy-guided transthoracic biopsy and bronchoscopic sampling in the diagnosis of pulmonary nodules. Orv. Hetil..

[3] Bhatt K.M., Tandon Y.K., Graham R., Lau C.T., Lempel J.K., Azok J.T., Mazzone P.J., Schneider E., Obuchowski N.A., Bolen M.A. (2018). Electromagnetic Navigational Bronchoscopy versus CT-guided Percutaneous Sampling of Peripheral Indeterminate Pulmonary Nodules: A Cohort Study. Radiology.

[4] Dale C.R., Madtes D.K., Fan V.S., Gorden J.A., Veenstra D.L. (2012). Navigational bronchoscopy with biopsy versus computed tomography-guided biopsy for the diagnosis of a solitary pulmonary nodule: A cost-consequences analysis. J. Bronchol. Interv. Pulmonol..

[5] Albertini R.E., Harrell J.H., Kurihara N., Moser K.M. (1974). Arterial hypoxemia induced by fiberoptic bronchoscopy. JAMA.

[6] Herbst R.S., Lippman S.M. (2007). Molecular signatures of lung cancer--toward personalized therapy. N. Engl. J. Med..

[7] Herbst R.S., Heymach J.V., Lippman S.M. (2008). Lung cancer. N. Engl. J. Med..

[8] Hsiao S.H., Chung C.L., Lee C.M., Chen W.Y., Chou Y.T., Wu Z.H., Chen Y.C., Lin S.E. (2013). Suitability of computed tomography-guided biopsy specimens for subtyping and genotyping of non-small-cell lung cancer. Clin. Lung Cancer.

[9] Brenner D.J., Hall E.J. (2007). Computed tomography—An increasing source of radiation exposure. N. Engl. J. Med..

[10] Henschke C.I., McCauley D.I., Yankelevitz D.F., Naidich D.P., McGuinness G., Miettinen O.S., Libby D.M., Pasmantier M.W., Koizumi J., Altorki N.K. (1999). Early Lung Cancer Action Project: Overall design and findings from baseline screening. Lancet.

[11] New York Early Lung Cancer Action Project Investigators (2007). CT Screening for lung cancer: Diagnoses resulting from the New York Early Lung Cancer Action Project. Radiology.

[12] Ng Y.L., Patsios D., Roberts H., Walsham A., Paul N.S., Chung T., Herman S., Weisbrod G. (2008). CT-guided percutaneous fine-needle aspiration biopsy of pulmonary nodules measuring 10 mm or less. Clin. Radiol..

[13] Wang B.Y., Hung J.J., Jeng W.J., Hsu W.H., Hsieh C.C., Huang M.H., Huang B.S., Liu J.S., Wu Y.C. (2010). Surgical outcomes in resected non-small cell lung cancer < or = 1 cm in diameter. J. Chin. Med. Assoc..

[14] Social Indicators 2009. Directorate General of Budget, Accounting and Statistics. https://ebook.dgbas.gov.tw/public/Data/331311345571.pdf.

[15] National Health Insurance The National Health Insurance Statistics 2009. https://www.nhi.gov.tw/english/Content_List.aspx?n=A8FFBD35C8B89CEB&topn=616B97F8DF2C3614.

[16] Tukey M.H., Wiener R.S. (2012). Population-based estimates of transbronchial lung biopsy utilization and complications. Respir. Med..

[17] Yeow K.M., See L.C., Lui K.W., Lin M.C., Tsao T.C., Ng K.F., Liu H.P. (2001). Risk factors for pneumothorax and bleeding after CT-guided percutaneous coaxial cutting needle biopsy of lung lesions. J. Vasc. Interv. Radiol..

[18] Wiener R.S., Schwartz L.M., Woloshin S., Welch H.G. (2011). Population-based risk for complications after transthoracic needle lung biopsy of a pulmonary nodule: An analysis of discharge records. Ann. Intern. Med..

[19] Laurent F., Montaudon M., Latrabe V., Begueret H. (2003). Percutaneous biopsy in lung cancer. Eur. J. Radiol..

[20] Moore E.H., Shepard J.A., McLoud T.C., Templeton P.A., Kosiuk J.P. (1990). Positional precautions in needle aspiration lung biopsy. Radiology.

[21] Tsukazan M.T.R., Vigo A., Silva V.D.D., Barrios C.H., Rios J.O., Pinto J.A.F. (2017). Lung cancer: Changes in histology, gender, and age over the last 30 years in Brazil. J. Bras. Pneumol..

[22] Krimsky W., Muganlinskaya N., Sarkar S., Vulchi M., Patel P., Rao S., Hammer J., Evans R., Qureshi M., Harley D. (2016). The changing anatomic position of squamous cell carcinoma of the lung—A new conundrum. J. Community Hosp. Int. Med. Perspect..

